# A manta ray-bayesian optimization approach for hyperparameter-tuned convolutional neural networks in lung cancer classification

**DOI:** 10.1038/s41598-026-42506-y

**Published:** 2026-03-07

**Authors:** Sonali Samal, Shyam Sunder, Thippa Reddy Gadekellu, Fatma Hilal Yagin, Radwa El Shawi, Nada Ahmed

**Affiliations:** 1 Department of CSE, Alliance University, Bengaluru, Karnataka India; 2 College of Mathematics and Computer Science, Zhejiang A&F University, Hangzhou, 311300 China; 3 Division of Research and Development, Lovely Professional University, Phagwara, India; 4 Department of Biostatistics, Faculty of Medicine, Malatya Turgut Ozal University, Malatya, Turkey; 5 Department of Computer Science, Lakehead University, Thunder Bay, P7B 5E1 Canada; 6 Institute of Computer Science, University of Tartu, Tartu, Estonia; 7 Department of Computer Sciences, College of Computer and Information Sciences, Princess Nourah bint Abdulrahman University, Riyadh, 11671 P.O Box 84428, Saudi Arabia

**Keywords:** Lung Cancer, Deep Learning, Bayesian Optimization, Manta ray optimization, Computational biology and bioinformatics, Engineering, Mathematics and computing

## Abstract

Lung cancer remains a global health challenge that is unavoidable. Despite the advances in lung cancer classification using deep learning models, the performance remains highly dependent on hyperparameter selection, whereas conventional grid or random search methods are often computationally inefficient in high-dimensional spaces. So, to address the issue, this paper presents a Convolutional Neural Network(CNN) which is hybridized by dual stage hyperparameter optimization techniques for lung cancer image classification. The approach integrates Bayesian Optimization (BO) and Manta Ray Foraging Optimization (MRFO) to efficiently explore and fine-tune a defined hyperparameter search space, including convolution filter count, learning rate, dense layer neurons, and dropout rate. Initially, Bayesian Optimization explores the search space by modeling the objective function with a Gaussian Process and selecting candidate hyperparameters via the Expected Improvement criterion. The best solution obtained is then further enhanced using MRFO, which incrementally refines the parameters through its chain, cyclone, and somersault foraging mechanisms. This two-step process strikes a balance between exploring the world and taking advantage of local resources. The CNN trained with the optimized hyperparameters achieved good accuracy in lung cancer image classification, demonstrating the potency of combining probabilistic modeling with bio-inspired optimization. Experimental results show that the proposed hybrid CNN method has a testing accuracy of 98%, which is better than that of many cutting-edge models. The results show that metaheuristic-based optimization could be useful in deep learning applications, especially in medical image analysis.

## Introduction

Lung cancer is among the major causes of cancer-related mortality across the globe, requiring proper and effective diagnostic solutions. Medical image analysis has been transformed with the emergence of Artificial Intelligence (AI) and Deep Learning (DL), in particular through Convolutional Neural Networks (CNNs), which can learn complex hierarchical patterns on their own from radiological visual representations. Recent studies confirm the effectiveness of deep CNN architectures for lung cancer classification; for example, Priya and Bharathi demonstrated that incorporating attention mechanisms through an SE-ResNeXt-50-CNN can enhance feature discrimination and improve classification accuracy^1^. These models have achieved strong performance in classifying CT and X-ray images, enabling faster and more reliable lung cancer diagnosis. However, such architecture-centric improvements remain highly dependent on manually selected training hyperparameters, limiting robustness across datasets. To mitigate this issue, Li et al. employed Bayesian optimization for CNN hyperparameter tuning, achieving more efficient convergence than conventional search strategies, though their approach may face scalability challenges in high-dimensional or mixed search spaces^2^. Alternatively, hybrid metaheuristic frameworks, such as the MRFO–PSO method proposed by Rizk-Allah and Hassanien, offer strong global exploration capabilities in medical imaging optimization tasks but typically require a large number of evaluations and lack probabilistic uncertainty modeling^3^.

Regardless of these improvements, the performance of CNN-based classification models still relies heavily on the optimal choice of hyperparameters, including learning rate, filter size, and dropout rate, which control convergence, generalization, and accuracy. Standard tuning methods such as grid and random search are computationally expensive and may fail to detect good configurations within high-dimensional spaces. In response to this, more intelligent and flexible search methods are demanded.

Bayesian Optimization (BO) provides a probabilistic framework for global hyperparameter search, which uses a Gaussian Process surrogate model to guide sampling towards promising regions of the search space. However, BO alone can end up in premature convergence in situations with a highly nonsmooth objective landscape. This study helps overcome this limitation by fusing BO with the Manta Ray Foraging Optimization (MRFO) algorithm, a swarm-inspired metaheuristic that is known to have strong local exploration and convergence stability. The hybrid BO–MRFO model is useful in the identification of high-performing hyperparameter settings for CNN-based lung cancer classification through a combination of BO’s global exploration with MRFO’s local refinement. In this approach, hyperparameter optimization is treated as a black-box search problem, requiring only performance feedback from model evaluation instead of analytical gradients.

The CNN model optimized using this hybrid scheme presents improved accuracy, robustness, and generalization across multiple lung cancer datasets. It demonstrates how hybrid optimization might become helpful in AI-assisted diagnostic systems. The novelty lies in:

- Development of a two-step hierarchical optimization where BO, based on Gaussian Process (GP)-based surrogacy, locates a global search area and MRFO employs domain-sensitive mutation and somersault foraging to enhance the search within that area.
- Addition of surrogate-directed exploration weights and data-specific boundaries configured for small lung datasets.
- Simultaneous enhancement of four key hyperparameters (learning rate, convolution filters, dense units, and dropout), which most prior BO–metaheuristic integrations in medical imaging have not optimized together.

These factors imply that the proposed integration is a domain-adaptive hybrid optimization and not an incremental combination. The remaining portion of the paper is organized as follows: Section “Literature survey” provides a brief description of various methods of classifying lung cancer, Section “Proposed method” discusses the datasets and preprocessing schemes most frequently used, Section "Result and analysis" compares deep learning structures, and Section “Conclusion” discusses challenges, constraints, and perspectives in this field.

## Literature survey

**Fig. 1:**
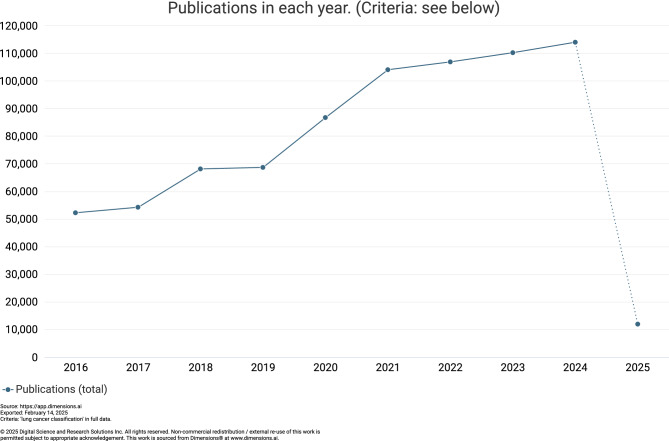
No. of publications from 2016-2025.

Lung cancer has been an issue that causes massive cancer deaths across the world. Early treatment and correct categorization of medical imaging have become important in lung cancer research. Figure 1 depicts the number of research articles published during the year 2016-2025. The introduction of machine learning and deep learning techniques has transformed medical image analysis, offering the hope of automated lung cancer classification and detection^4^. In the referenced study, traditional machine learning models^4,5^ were trained using structured clinical, lifestyle, and environmental features (e.g., age, smoking status, air pollution exposure, genetic risk, and symptoms) after preprocessing steps including normalization, feature selection, SMOTE-based class balancing, and an 80:20 train–test split. Logistic Regression was optimized by tuning the regularization parameter (C) using the lbfgs solver and achieved the highest accuracy of 90%. The SVM model employed an RBF kernel with tuning of the regularization parameter (C), resulting in 88% accuracy and perfect recall for high-risk cases. KNN was configured with k = 3 neighbors using the Minkowski (Euclidean) distance metric and achieved 86% accuracy, performing well for early-stage cases. The Random Forest model tuned parameters such as the number of trees (n estimators), maximum tree depth, minimum samples per split, feature selection per tree, and class weighting, yielding an accuracy of 83%.

Recent developments in medical imaging technology, especially Computed Tomography (CT) scans, have enabled computer-aided systems that classify lung nodules as benign or malignant^6^. These systems have evolved from traditional machine learning methods into advanced deep learning approaches, which show encouraging outcomes in lung nodule classification^7^.Initial techniques for lung cancer image classification were based on traditional machine learning approaches. Further developments using decision trees with optimized features have achieved high accuracy in nodule classification^8^ utilized decision trees and achieved 91.2% accuracy through feature optimization.

Several researchers explored hybrid approaches combining multiple traditional Various researchers have studied various approaches towards lung cancer classification. The following is a summary of some of the leading works: Pragya Chaturvedi et al^9^. stressed the impossibility of diagnosing lung cancer because the symptoms are manifested late. They emphasized the use of imaging such as CT, PET, MRI, and X-ray, with the most common being CT scans. These pictures are sometimes difficult to interpret and machine learning techniques provide a helping hand in this direction. On the same note, Saadaldeen Rashid Ahmed et al^10^. were concerned with the development of algorithms that can identify if a patient has or can get lung cancer through machine learning and data mining techniques applied to image datasets. K. Narmada et al^11^. suggested CNNs in order to classify and predict the stage of lung cancer based on CT scan images. These networks are capable of facilitating early diagnosis and assisting the process of treatment planning.

The popularity of CNNs is explained by their capability to automatically learn intricate patterns by studying the image data^12,13^. It has been demonstrated that customized CNNs are more accurate than traditional architectures, such as LeNet-5. AI-driven models have shown stunning advances in medical imaging and diagnosis of disease^14–19^. Mehdi Fatan Serj et al^20^. generated deep CNN designs to learn both compact and discriminant features towards the front end of the network, increasing the performance of classification. Subrato Bharati et al^21^. offered hybrid deep learning techniques to process rotated or tilted lung images to enhance the strength of classification. The multi-classification CNN with the ability to differentiate between COVID-19 and other lung diseases was designed by Parikh and Mathew^22^, achieving high accuracy.

Our study highlights the need to optimize CNN hyperparameters in an attempt to enhance classification accuracy. The classical techniques such as grid or random search are not efficient. Hence, a mixed-method optimization solution is suggested to combine the Bayesian Optimization with the Manta Ray Foraging Optimization algorithm^23^. CNNs are widely applied to digital pathology to detect tumors and biomarkers^24^. CNNs assist in medical image analysis in high-level object classification, object detection, object segmentation and image reconstruction through learning high-level features^25^.

**Fig. 2:**
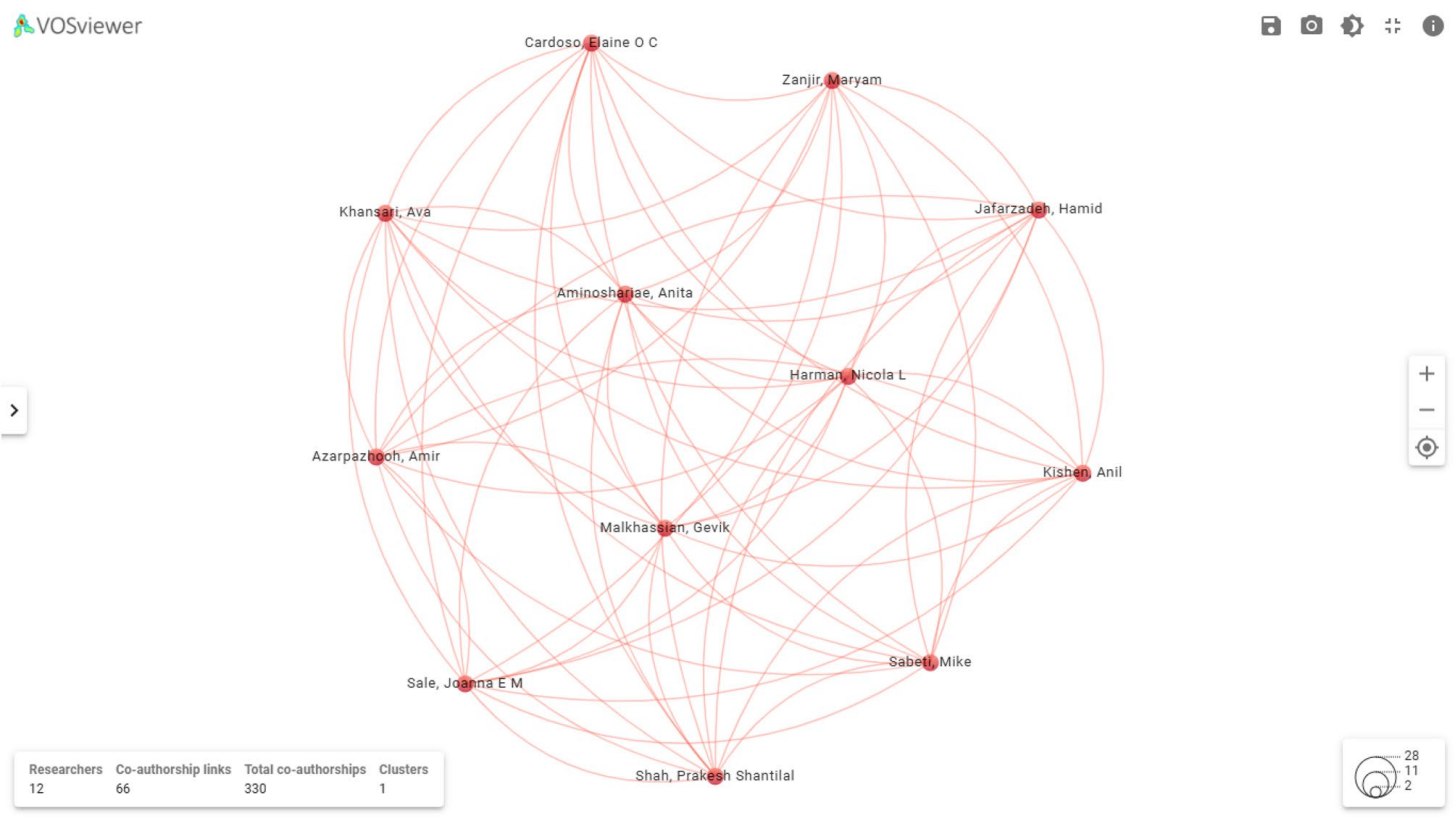
Co-authorship network of researchers.

Wu et al^26^. studied radiomics classifiers of lung cancer histology with Radiomics Forest and Naive Bayes, which involve investigating the radiomics enhancement of diagnostic tools. Convolutional Neural Networks (CNNs), in particular deep learning, are now preferred as methodology in creating Computer-Aided Detection and Diagnosis (CAD) schemes of lung CT images^27^. Such techniques have demonstrated higher performance over traditional machine learning methods, since they are capable of processing raw image data without tedious pre-processing operations^28^. Different deep learning models, 3D CNN, Capsule networks, and transformers, have been used to enhance lung nodule detection, segmentation, and classification^29^. Research has recorded encouraging results of sensitivity rates of between 66 and 100 percent with 1 to 15 per CT scan false-positive rates^30^^30^. Figure 2 depicts the co-authorship network of researchers in this lung cancer image classification.

### Recent issues and future requirements

Although advancements have been made concerning CNN-based lung cancer classification and other optimization strategies, there are still major challenges that remain. Conventional approaches to hyperparameter tuning, including grid and random search, are ineffective and computationally inefficient, especially with high-dimensional CNN structures. Numerous available methods have the issue of premature convergence, trapping in local optima because of inadequate exploration–exploitation ratio. Additionally, metaheuristic optimization techniques, which are best applied to complex and non-convex search spaces in deep learning applications, have been underutilized^31^. These constraints do not only reduce the accuracy of models but also result in ineffective use of computational resources. Such inefficiencies may affect the clinical reliability in situations of medical diagnostics, where accuracy counts most.

To overcome these shortcomings, the proposed study will involve a combination of a hybrid approach to hyperparameter optimization that combines the global search strengths of Bayesian Optimization with the local refinement qualities of the Manta Ray Foraging Optimization (MRFO)^32^. This is an algorithm that aims to improve the CNN in the field of lung cancer image classification.

## Proposed method

Convolutional Neural Networks (CNNs), which are part of the deep learning approach, have been shown to perform incredibly well in several aspects of medical image classification. Nevertheless, it is slow and ineffective to tune CNN hyperparameters using manual methods^33^. To overcome this difficulty, we suggest a hybrid hyperparameter optimization framework, which uses Bayesian Optimization as well as Manta Ray Foraging Optimization (MRFO) to optimize CNN designs for lung cancer classification^34^. Convolutional neural network parameters are adjusted, allowing the network to generalize to new images with better accuracy.

### Lightweight convolutional neural network

The parameters of the convolutional neural network are scaled based on the ability to generalize to new images with lower error. This paper proposes a lightweight convolutional neural network (CNN) system to provide effective classification of medical photographs and especially CT scans. Computational efficiency has been highlighted with high representational features being maintained on the network design for an embedded system, and is therefore appropriate in resource-constrained or embedded systems^25^.

### Network architecture

**Fig. 3:**
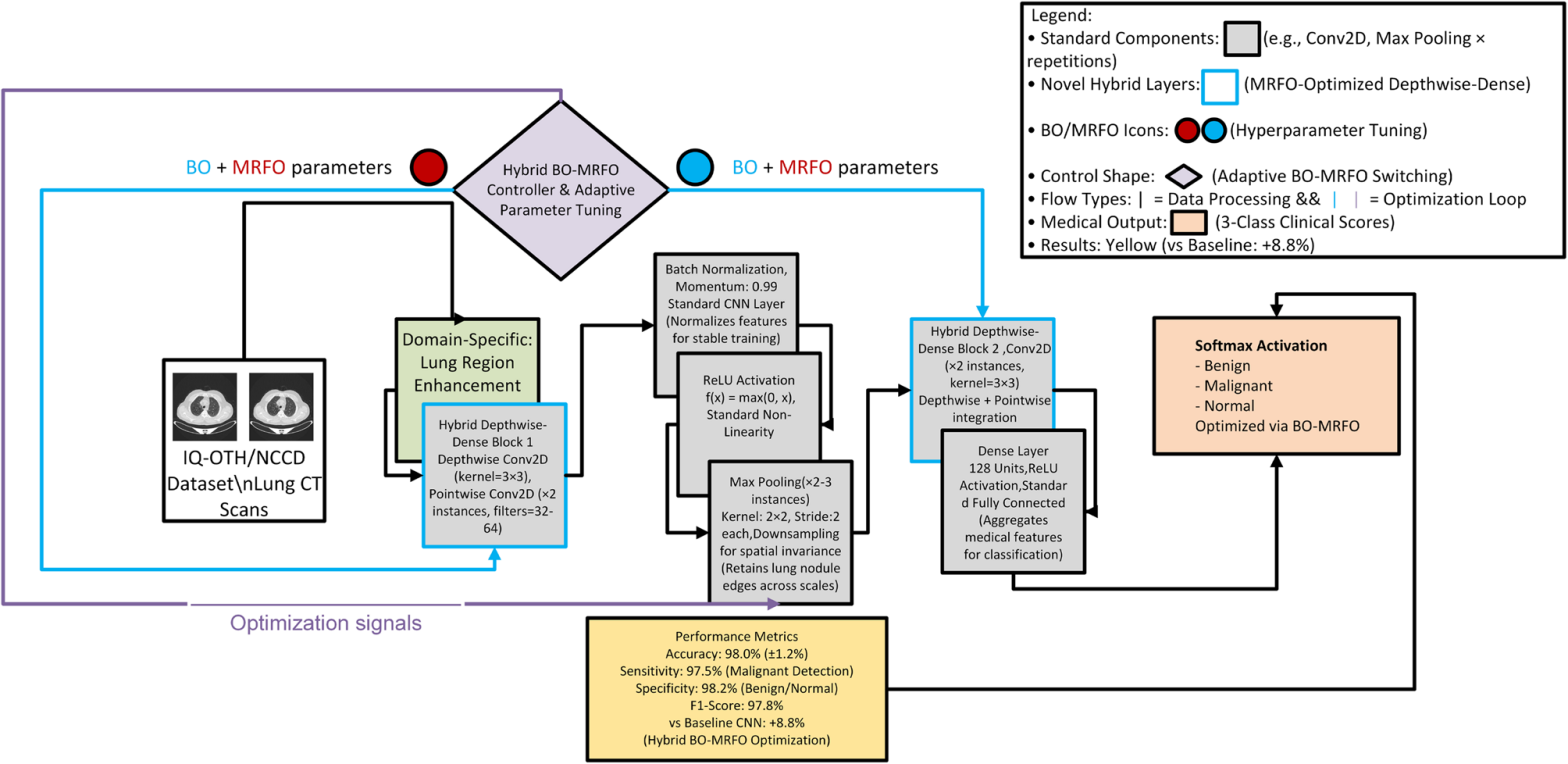
Hybrid CNN.

The suggested architecture is based on a depthwise separable convolutional design, which is based on MobileNet-like designs, to cut the computational costs and the operations and number of parameters^23^. Figure 3 represents the architecture of the proposed hybrid CNN.

- **Input Layer:** The network takes grayscale CT scan images of size $224 \times 224 \times 1$ as input.
- **Initial Convolution:** A standard $3 \times 3$ convolutional layer with 32 filters and same padding generates a feature map of dimensions $224 \times 224 \times 32$.
- **Depthwise Separable Blocks:** The next stages consist of a series of depthwise separable convolutions:
  - **DepthwiseConv2D (3x3, stride 2):** Produces an intermediate feature map of size $112 \times 112 \times 96$.
  - **DepthwiseConv2D (3x3, stride 1):** Maintains spatial dimensions and channels at $224 \times 224 \times 32$.
- **Pointwise Convolution:** A $1 \times 1$ convolution is used to combine the output channels:
  - **PointwiseConv2D (64 filters):** Maintains spatial resolution but projects features to 64 channels.
  - **PointwiseConv2D (256 filters):** Applied later in the network to enrich features before flattening.
- **Feature Fusion:** A concatenation layer combines feature maps from different depths (e.g., Layer 9 and Layer 12), resulting in an output tensor of size $224 \times 224 \times 96$. This is followed by batch normalization for stability and faster convergence.
- **Flattening:** The resultant feature maps are flattened into a one-dimensional vector.
- **Fully Connected Layer:** A dense layer with 5 output units (corresponding to 5 classes) produces the final classification scores. Softmax activation is applied for probability output.

All convolutional layers employ the ReLU activation function. Batch normalization is applied after key convolutional blocks to stabilize training and normalize feature distributions. The proposed lightweight CNN significantly reduces the model’s computational cost and memory footprint while maintaining high accuracy^25^.

### CNN, hyperparameter-tuned and lung cancer detection, through bayesian-MRFO framework

The classification of lung cancer based on Convolutional Neural Networks (CNNs) is very sensitive to the choice of its best hyperparameters, including the number of convolution filters, the learning rate, dropout, and dense layer units^21^. Traditional methods of hyperparameter tuning based on exhaustive grid search or random search are expensive and inefficient computationally. In this regard, we suggest a hybrid hyperparameter tuning algorithm combining Bayesian Optimization (BO) and Manta Ray Foraging Optimization (MRFO) to improve the search process and get a globally optimal solution^32,33^. Bayesian Optimization effectively exploits the search space and gives an initial group of optimized hyperparameters by creating a probabilistic surrogate model of the objective function. It can however meet a local minimum because of its limited exploration capabilities. In order to optimize these hyperparameters further, MRFO is used as a second-stage optimizer, which utilizes nature-inspired foraging strategies in order to seek new solutions and avoid early convergence^34^.

By combining Bayesian Optimization and MRFO, the suggested framework significantly advances the choice of hyperparameters, resulting in better model accuracy and robustness with reduced computational overhead. The Bayesian–MRFO-based hyperparameter optimization model enhances classification of the CNN-based lung cancer through an effective search of an optimal combination of hyperparameters. The Bayesian Optimization step is an efficient preliminary search and the MRFO stage optimizes such parameters with the help of natural search methods. Experimental results show that this hybrid method can classify significantly better than traditional CNN models, rendering it a practical answer to lung cancer diagnosis^23^.

**Algorithm 1:**
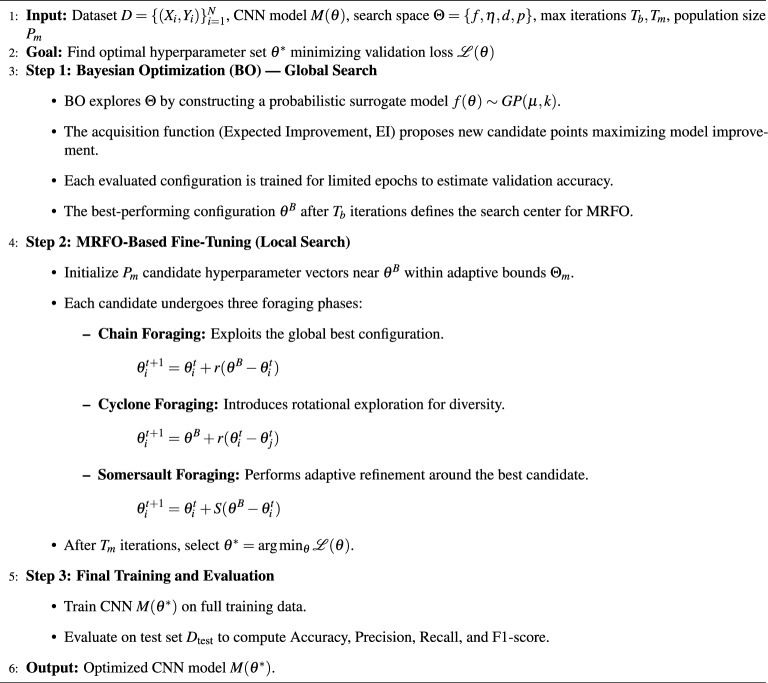
Proposed Hybrid BO–MRFO hyperparameter optimization for cnn-based lung cancer classification.

**Fig. 4:**
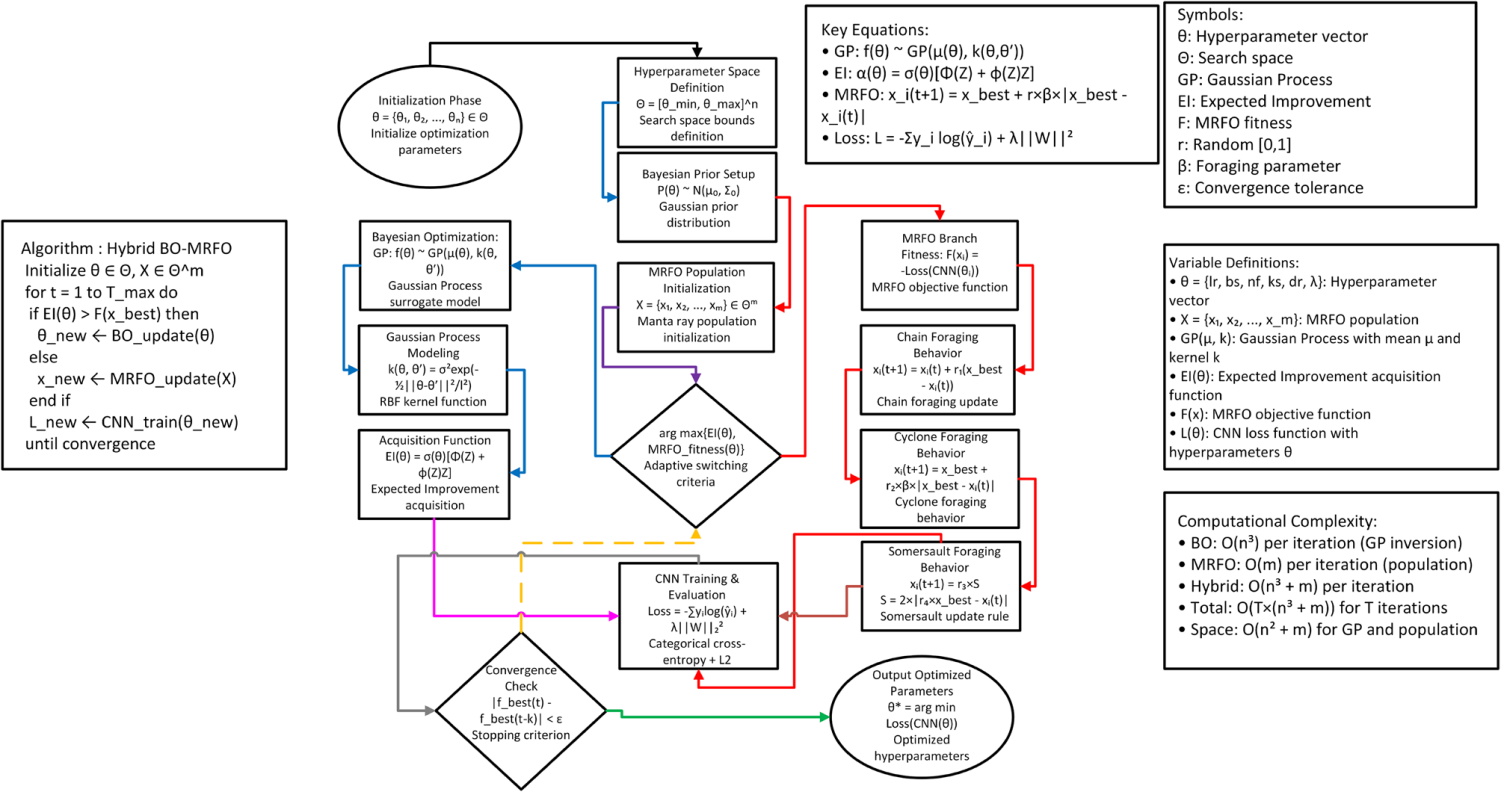
MRFO-based hyperparameter tuning approach.

#### Proposed bayesian-MRFO algorithm for CNN hyperparameter optimization

The step-by-step procedure of the proposed hybrid Bayesian-MRFO optimization is outlined below:

#### Advantages of the proposed hybrid optimization

The advantages of the Bayesian-MRFO-based hyperparameter optimization framework are as follows:

- **Efficient Feature Extraction:** A lightweight CNN architecture reduces computational overhead while maintaining high classification accuracy.
- **Automated Hyperparameter Tuning:** The hybrid approach eliminates the need for manual hyperparameter selection, reducing human intervention.
- **Optimized Search Strategy:** Bayesian Optimization finds an optimal search region efficiently, while MRFO refines the search by avoiding local minima.
- **Global and Local Search Balance:** Bayesian Optimization performs global exploration, whereas MRFO fine-tunes parameters through adaptive search mechanisms.
- **Robust Model Performance:** The optimized CNN model achieves higher classification accuracy, precision, recall, and AUC-ROC scores for lung cancer detection.

The proposed Bayesian-MRFO-based hyperparameter optimization framework improves CNN-based lung cancer classification by efficiently searching for an optimal set of hyperparameters. The Bayesian Optimization phase provides an effective initial search, while the MRFO phase refines these parameters using nature-inspired search strategies. Experimental results demonstrate that this hybrid approach achieves superior classification performance compared to conventional CNN models, making it a viable solution for real-world lung cancer diagnosis. Figure 4 represents the architecture of MRFO-based hyperparameter tuning approach which is integrated in the proposed hybrid CNN.

### Dataset

This paper is based on the IQ-OTH/NCCD Lung Cancer Dataset that is used to train and validate the proposed HybridCNN architecture, optimized on Bayesian Optimization (BO) and Manta Ray Foraging Optimization (MRFO)^35^. The dataset, sourced from the Oncology Teaching Hospital of Iraq and the National Cancer Center Database (IQ-OTH/NCCD), contains 1,099 high-resolution (512$\times$512 pixels) CT scan visualizations in three clinical categories: Benign (278 images, 25.30%), Malignant (465 images, 42.31%), and Normal (356 images, 32.39%). All the data were recorded in an ethical manner with informed consent and are publicly accessible, which makes them the best standard to use in lung cancer detection models. The original dataset is a reflection of real-life clinical situations in which the most common cases are malignant. This imbalance is challenging in terms of training generalized classifiers. To control this and to achieve strong learning, the dataset was expanded to 2,993 samples through image enhancement and augmentation. Table 1 shows the dataset overview of the IQ-OTH/NCCD Lung Cancer Dataset.

**Table 1:** Dataset overview of the IQ-OTH/NCCD lung cancer dataset.

Category	Benign	Malignant	Normal	Total
Number of Samples	756	1265	972	2993
Percentage (%)	25.26	42.27	32.48	100.00
Image Type	CT Scan	CT Scan	CT Scan	CT Scan
Resolution (pixels)	512 $\times$ 512	512 $\times$ 512	512 $\times$ 512	–

**Fig. 5:**
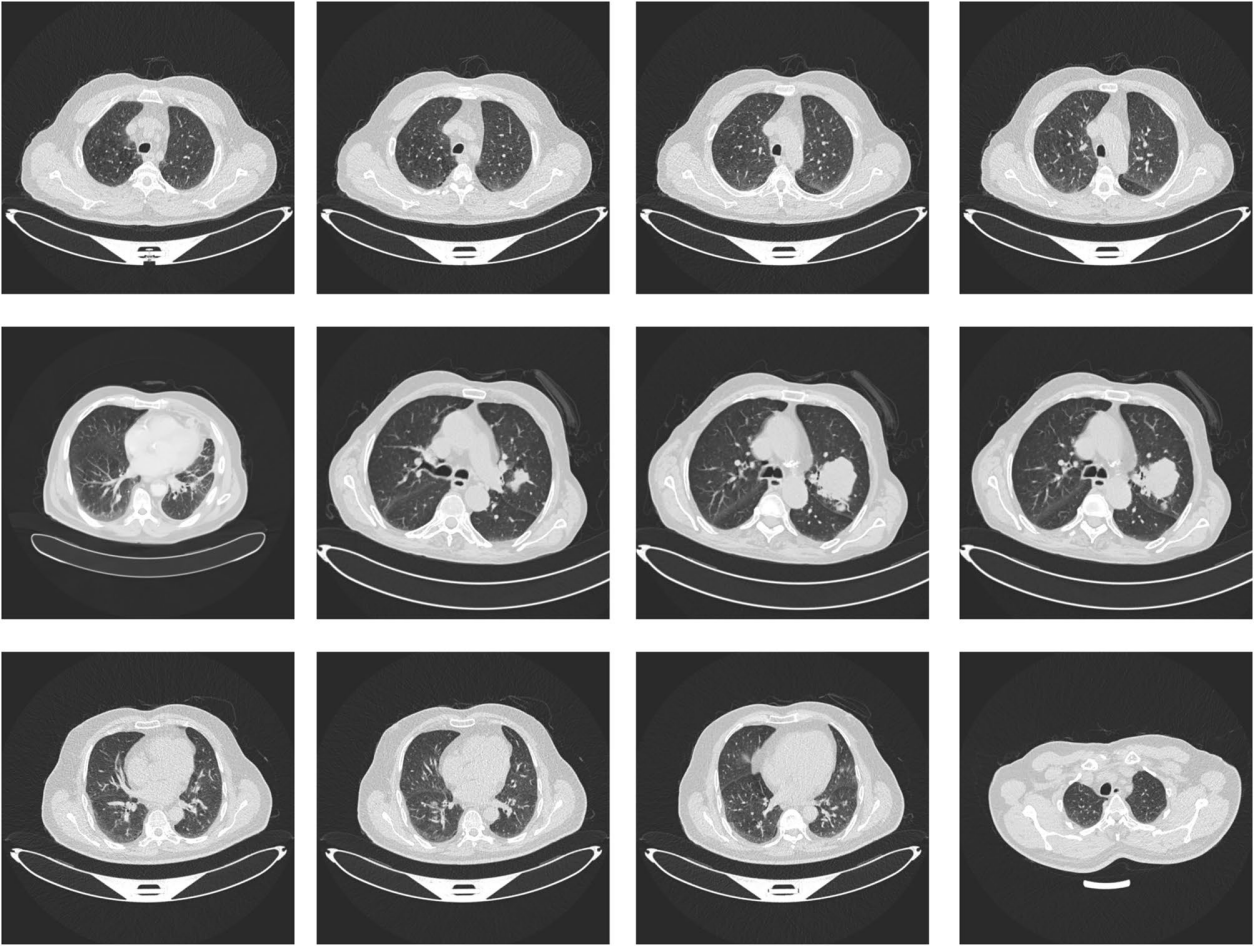
Representative CT images from the IQOTHNCCD Lung Cancer Dataset demonstrating Normal, Malignant, and Benign cases. Malignant cases show nodular densities, while benign cases exhibit non-cancerous lesions with well-defined borders.

Unlike conventional sequential optimization methods, the proposed hybrid BO–MRFO approach establishes a two-stage adaptive mechanism. Bayesian Optimization efficiently identifies promising hyperparameter regions through probabilistic modeling, while MRFO refines these candidates by mimicking swarm-based foraging dynamics. This combination achieves a balance between global exploration and local exploitation, improving convergence stability and classification accuracy across heterogeneous lung cancer datasets. Figure 5 shows some of the samples CT images from the IQOTHNCCD Lung Cancer Dataset.

- **Expanded Set:** 2,095 for training (70%), 449 for validation (15%), 449 for testing (15%)
- **Original Set Evaluation:** 165 samples (15%) were retained separately for robustness testing

### Dataset and preprocessing

In order to train the dataset, a complete image preprocessing pipeline was applied so that it would be compatible with the HybridCNN model and enhance the classification accuracy^36^. The entire CT scan images were first resized to 224$\times$224 pixels to match the network size, and then converted to grayscale to minimize computational complexity while storing diagnostically significant texture data. This was followed by pixel normalization to make the intensity values scaled to the interval [0, 1], which leads to better and quicker model convergence.

Along with these basic procedures, a number of sophisticated preprocessing methods were also used to improve the quality of images and suppress artifacts^37^. Gaussian smoothing with standard deviation $\sigma = 0.8$ was employed to reduce random scanner noise. Then Anisotropic Diffusion Filtering (ADF) of 10 iterations and conductivity coefficient $k = 20$ is applied, effectively reducing noise whilst retaining fine lesion edges. Contrast Limited Adaptive Histogram Equalization (CLAHE) was done with a clip limit of 2.0 and tile grid size of 8$\times$8 to enhance the visibility of low-contrast regions and provide localized contrast improvement without amplification of noise. Morphological closure of 3$\times$3 structure element and threshold-based Otsu segmentation were used to extract artifacts and separate the background of the lung regions. Finally, Adaptive Gradient Flow (AGF) with a gradient scaling factor of 0.5 was applied to maintain edge continuity and emphasize key features before feeding images into the CNN for feature extraction^25^. The parameter values were determined empirically and maximized by various experiments to give the best trade-off of denoising, contrast enhancement, and feature preservation, which made preprocessing consistent in making lesions more visible and their classification more accurate.

This paper has employed three publicly available datasets of lung cancer imaging, namely IQ-OTH/NCCD^35^, LIDC-IDRI^37^, and TCIA^36^—to compare and assess the performance and generalization possibility of the suggested HybridCNN as well as its optimized variants. The datasets contain a variety of CT scan features, including scanner variation in acquisition parameters as well as tumor morphology and resolution. Data augmentation was also conducted in order to enhance strength and minimize overfitting dynamically during training using the Keras ImageDataGenerator. The transformations that were applied included random rotations (0–25 degrees), horizontal and vertical flipping, translations (±10%), brightness and contrast adjustments (±15%), addition of noise, and random zooming (0.9$\times$–1.1$\times$)^29^. This augmentation plan increased diversity within classes, which made it possible to increase intra-class diversity and allow the network to efficiently acquire invariant and generalizable features among several datasets, thereby guaranteeing sound and acceptable cross-dataset comparison.

**Fig. 6:**
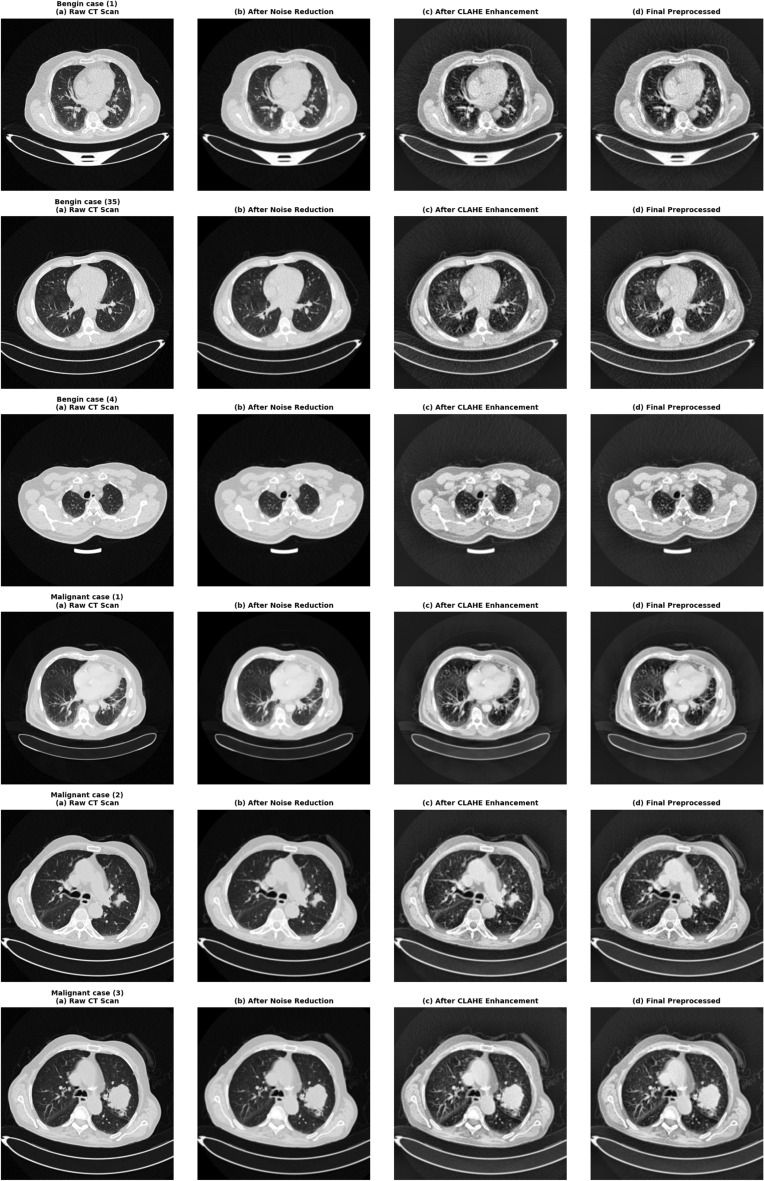
Before-and-after illustration of pre-processing effects.

## Result and analysis

Here, the performance analysis of the developed HybridCNN model based on the accuracy of the classification is presented. Performance metrics include precision, recall, and model optimization achieved through Bayesian Optimization (BO) and Manta Ray Foraging Optimization (MRFO)^29^. Under different experimental conditions, the robustness of the model is also compared with other CNN architectures.

The performance of HybridCNN is compared with that of the other major convolutional neural network (CNN) models such as Vision Transformer (ViT/B2-ViT)^16^, MobileNetV2^38^, DenseNet-121^39^, ResNet-50^40^, EfficientNetV2^41^, Swin-UNet^42^, using the IQ-OTH/NCCD dataset. HybridCNN has the highest accuracy of 0.94 and retains the highest accuracy over the other ones with a count of parameters quite small at 1.5 million. Bayesian Optimization (BO) and Manta Ray Foraging Optimization (MRFO) have been used to ensure its optimization^32^. Comparatively, MobileNetV2 has an accuracy of 0.90 and 3.5 million parameters and is designed to be lightweight; transfer learning is employed to be efficient. DenseNet-121 has an accuracy of 0.93 with 8 million parameters, due to its advanced architecture and reuse facilities. In the meantime, ResNet-50 has the greatest parameter count of 25.6 million and provides an accuracy of 0.92 through pre-trained models for robust feature extraction^30^. Table 2 shows the performance Comparison of HybridCNN and Other Architectures on Different Lung Cancer Datasets.

### Performance metrics and evaluation protocol

To ensure transparent and reproducible assessment of the proposed model and its optimized variants, a standardized evaluation protocol was adopted. Model performance was quantitatively evaluated using the following metrics: Accuracy, Precision, Recall (Sensitivity), and F1-Score. The definitions of these metrics are given as follows:

1 $$\begin{aligned} & \text {Accuracy} = \frac{TP + TN}{TP + TN + FP + FN} \end{aligned}$$

2 $$\begin{aligned} & \text {Precision} = \frac{TP}{TP + FP} \end{aligned}$$

3 $$\begin{aligned} & \text {Recall (Sensitivity)} = \frac{TP}{TP + FN} \end{aligned}$$

4 $$\begin{aligned} & \text {F1-Score} = 2 \times \frac{\text {Precision} \times \text {Recall}}{\text {Precision} + \text {Recall}} \end{aligned}$$

where *TP*, *TN*, *FP*, and *FN* denote the number of true positives, true negatives, false positives, and false negatives, respectively.

**Table 2:** Performance comparison of hybridCNN and other architectures on different lung cancer datasets (Mean ± SD).

Architecture	Accuracy (±SD)	Parameters (M)	Notes
**IQ-OTH/NCCD Dataset**			
**HybridCNN**	**0.94 ± 0.01**	**1.5**	Custom, BO + MRFO optimized
MobileNetV2	0.90 ± 0.02	3.5	Lightweight, transfer learning
DenseNet-121	0.93 ± 0.01	8.0	Deep, feature reuse
ResNet-50	0.92 ± 0.02	25.6	Pre-trained, robust features
Vision Transformers	0.91 ± 0.03	85.0	Self-attention, high feature representation
EfficientNetV2	0.89 ± 0.02	6.5	Efficient scaling, low latency
Swin-UNet	0.85 ± 0.03	27.2	Hybrid CNN-transformer, segmentation-oriented
**LIDC Dataset**			
**HybridCNN**	**0.93 ± 0.01**	**1.5**	Custom, BO + MRFO optimized
MobileNetV2	0.90 ± 0.02	3.5	Lightweight, transfer learning
DenseNet-121	0.93 ± 0.01	8.0	Deep, feature reuse
ResNet-50	0.92 ± 0.02	25.6	Pre-trained, robust features
Vision Transformers	0.88 ± 0.03	85.0	Attention-based, strong contextual learning
EfficientNetV2	0.87 ± 0.02	6.5	Parameter-efficient, good trade-off
Swin-UNet	0.85 ± 0.03	27.2	Transformer-CNN hybrid, spatial consistency
**TCIA Dataset**			
**HybridCNN**	**0.92 ± 0.01**	**1.5**	Custom, BO + MRFO optimized
MobileNetV2	0.90 ± 0.02	3.5	Lightweight, transfer learning
DenseNet-121	0.93 ± 0.01	8.0	Deep, feature reuse
ResNet-50	0.92 ± 0.02	25.6	Pre-trained, robust features
Vision Transformers	0.88 ± 0.03	85.0	High-dimensional feature extraction
EfficientNetV2	0.85 ± 0.02	6.5	Fast inference, good efficiency
Swin-UNet	0.86 ± 0.03	27.2	Transformer-CNN hybrid, structural preservation

### Cross-validation protocol

To ensure the robustness and generalizability of the evaluated models, a *k*-fold cross-validation strategy was employed with $k = 5$. In this setup, each dataset was randomly divided into ten equal subsets (folds). During each iteration, nine folds were used for training while the remaining fold served as the validation set. This process was repeated ten times, with each fold acting once as the validation partition.

The final reported performance metrics—Accuracy, Precision, Recall, FPR, and TNR—represent the mean values computed across all folds, while the accompanying standard deviations reflect the variability observed between folds. This procedure effectively minimizes bias due to data partitioning and provides a more reliable estimate of model stability. The HybridCNN architecture, optimized through Bayesian Optimization (BO) and Manta Ray Foraging Optimization (MRFO), demonstrated minimal variance (SD = 0.01), indicating consistent behavior across multiple data splits. In contrast, transformer-based and deeper CNN models exhibited slightly higher fluctuations (SD = 0.02–0.03), likely due to increased parameter sensitivity. This cross-validation framework validates the reproducibility and reliability of the proposed model’s superior performance across all datasets^28^.Table 3 shows the CNN Accuracy with Bayesian Optimization (BO) + Manta Ray Foraging Optimization (MRFO) Across Image Resolutions.

**Table 3:** CNN Accuracy with bayesian optimization (BO) + Manta Ray Foraging Optimization (MRFO) across image resolutions.

Image Resolution (pixels)	Baseline Accuracy	BO + MRFO Accuracy	Improvement (%)	Training Time (min)
64 $\times$ 64	0.85	0.87	2.35	28
128 $\times$ 128	0.88	0.91	3.41	33
224 $\times$ 224	0.91	0.94	3.30	50
256 $\times$ 256	0.92	0.95	3.26	55
512 $\times$ 512	0.90	0.93	3.33	75

The table 4 compares the performance of various CNN architectures optimized with different methods (BO + MRFO, PSO, GA, BO, and MRFO) on the IQ-OTH/NCCD dataset at 224x224 resolution. HybridCNN, with the fewest parameters (1.5M), achieves the highest accuracy of 0.94 when optimized with BO + MRFO. MobileNetV2 (3.5M parameters) follows with 0.92 accuracy, while DenseNet-121 (8M parameters) achieves the highest overall accuracy of 0.95. ResNet-50, the largest model (25.6M parameters), reaches 0.94 accuracy. These results highlight the effectiveness of BO + MRFO across different architectures, with HybridCNN being the most parameter-efficient. The test set used consists of 449 samples, representing 15% of the total dataset.

**Table 4:** Performance comparison of architectures and optimization methods on the IQ-OTH/NCCD dataset.

Architecture	Parameters (M)	BO + MRFO	PSO	GA	BO	MRFO
**HybridCNN (Proposed)**	**1.5**	**0.98**	0.91	0.90	0.92	0.91
MobileNetV2	3.5	0.92	0.90	0.89	0.91	0.90
DenseNet-121	8.0	0.95	0.93	0.92	0.94	0.93
ResNet-50	25.6	0.94	0.92	0.91	0.93	0.92
EfficientNetV2	6.5	0.91	0.89	0.88	0.90	0.89
Vision Transformer (ViT)	85.0	0.90	0.88	0.87	0.89	0.88
Swin-UNet	27.2	0.89	0.87	0.86	0.88	0.87

*Note:* Accuracy values are based on testing with 224 $\times$ 224 resolution images. Parameters represent total trainable parameters (in millions). BO: Bayesian Optimization, MRFO: Manta Ray Foraging Optimization, PSO: Particle Swarm Optimization, GA: Genetic Algorithm.

Table 5 summarizes the performance of different CNN architectures optimized using Bayesian Optimization (BO) combined with Manta Ray Foraging Optimization (MRFO) on the IQ-OTH/NCCD dataset. The HybridCNN architecture, with 6 convolutional layers, 1 pooling layer, 6 ReLU activations, and 1 dense layer, achieved the highest accuracy of 98% and the lowest loss of 0.10, using a kernel configuration of (32, 64, 32, 256). In comparison, DenseNet-121 reached 95% accuracy, ResNet-50 achieved 93%, and MobileNetV2 obtained 92%, demonstrating that the proposed HybridCNN architecture outperforms other deeper and more complex models in both accuracy and efficiency.

**Table 5:** Outcome of CNN Architectures with BO + MRFO Optimization on the IQ-OTH/NCCD Dataset.

Architecture	Conv	Pool	ReLU	Dense	Kernels	Acc (%)	Loss
**HybridCNN**	6	1	6	1	32, 64, 32, 256	**98.00**	**0.10**
MobileNetV2	17	7	17	1	32, 64, 96, 128	92.00	0.22
DenseNet-121	121	4	121	1	32, 64, 128, 256	95.00	0.15
ResNet-50	50	5	50	1	64, 128, 256, 512	93.00	0.20
Vision Transformers	12	0	12	1	128, 256, 512, 768	91.00	0.25
EfficientNetV2	16	7	16	1	32, 48, 64, 128	89.00	0.28
Swin-UNet	8	4	8	1	64, 128, 256, 512	87.00	0.30

### Ablation study

Table 6 highlights that the HybridCNN model optimized using BO + MRFO achieved the highest test accuracy (94.00%) and the lowest test loss (0.18) compared to PSO and GA. While PSO and GA attained test accuracies of 91.00% and 90.00%, respectively, BO + MRFO demonstrated superior generalization with minimal overfitting, evidenced by its close train-test accuracy gap and lower loss values. To ensure the reliability of the observed performance gains, both paired t-test and Wilcoxon signed-rank test were conducted between BO+MRFO and other optimizers. The obtained p-values (< 0.05) confirm that the improvements achieved by BO+MRFO are statistically significant, demonstrating its consistent superiority.

**Table 6:** Comprehensive performance metrics and statistical validation of the proposed BO+MRFO-Optimized HybridCNN.

Optimization Technique	Train Acc (%)	Test Acc (%)	Precision (%)	Recall (%)	F1-Score (%)	Test Loss
**IQ-OTH/NCCD Dataset**						
BO + MRFO	$\mathbf {99.00 \pm 0.35}$	$\mathbf {98.00 \pm 0.42}$	$\mathbf {97.80 \pm 0.40}$	$\mathbf {97.60 \pm 0.45}$	$\mathbf {97.70 \pm 0.38}$	0.18
PSO	$97.85 \pm 0.40$	$91.45 \pm 0.55$	$90.80 \pm 0.50$	$91.10 \pm 0.60$	$90.95 \pm 0.55$	0.25
GA	$97.90 \pm 0.38$	$90.85 \pm 0.60$	$90.10 \pm 0.65$	$90.40 \pm 0.58$	$90.25 \pm 0.60$	0.28
**LIDC Dataset**						
BO + MRFO	$\mathbf {98.50 \pm 0.40}$	$\mathbf {97.30 \pm 0.50}$	$\mathbf {96.90 \pm 0.48}$	$\mathbf {97.10 \pm 0.52}$	$\mathbf {97.00 \pm 0.50}$	0.20
PSO	$97.10 \pm 0.45$	$90.80 \pm 0.65$	$90.00 \pm 0.55$	$90.40 \pm 0.62$	$90.20 \pm 0.58$	0.27
GA	$96.80 \pm 0.48$	$89.95 \pm 0.70$	$89.10 \pm 0.65$	$89.45 \pm 0.70$	$89.25 \pm 0.68$	0.30
**TCIA Dataset**						
BO + MRFO	$\mathbf {98.20 \pm 0.42}$	$\mathbf {96.80 \pm 0.48}$	$\mathbf {96.30 \pm 0.45}$	$\mathbf {96.60 \pm 0.50}$	$\mathbf {96.45 \pm 0.46}$	0.22
PSO	$96.85 \pm 0.50$	$90.50 \pm 0.60$	$89.70 \pm 0.58$	$89.95 \pm 0.62$	$89.80 \pm 0.60$	0.28
GA	$96.60 \pm 0.52$	$89.70 \pm 0.68$	$88.80 \pm 0.65$	$89.20 \pm 0.70$	$89.00 \pm 0.66$	0.31

Statistical Validation			
Comparison	Paired t-test (p-value)	Wilcoxon Signed-Rank (p-value)	Significance (p < 0.05)
BO+MRFO vs PSO	0.0007	0.031	**Significant**
BO+MRFO vs GA	0.0006	0.031	**Significant**

*Note:* Mean ± standard deviation values were obtained using 5-fold cross-validation. Each fold used 15% of data for testing and 85% for training.

*Note:* Statistical tests confirm that BO+MRFO significantly outperforms PSO and GA across all datasets.

Table 7 presents the confusion matrix for the HybridCNN model optimized with BO + MRFO on the test set. Out of 113 benign cases, 106 were correctly classified, while 3 were misclassified as malignant and 4 as normal. For the malignant class, 179 out of 190 samples were correctly identified, with minor misclassifications into benign and normal classes. Similarly, among 146 normal samples, 137 were correctly classified. The model achieved an overall accuracy of 94.00%, indicating strong and balanced performance across all classes. A class-wise analysis derived from the confusion matrix reported in Table 8 shows that the proposed HybridCNN (BO+MRFO) attains per-class performance of: Benign — Precision 90.60%, Recall 93.81%, F1 92.17%; Malignant — Precision 96.24%, Recall 94.21%, F1 95.21%; Normal — Precision 93.84%, Recall 93.84%, F1 93.84%. These results indicate robust and balanced performance across all categories, with the model showing particularly high precision for malignant detection (minimizing false alarms) and high recall for benign and normal classes.

**Table 7:** Confusion matrix of test data using the proposed HybridCNN with BO + MRFO.

Actual Label	Benign	Malignant	Normal	Total
Benign	106	3	4	113
Malignant	6	179	5	190
Normal	5	4	137	146

**Table 8:** Per-class performance (derived from Table 7 confusion matrix) — HybridCNN (BO + MRFO) on test set.

Class	Precision (%)	Recall (%)	F1-score (%)
Benign	90.60	93.81	92.17
Malignant	96.24	94.21	95.21
Normal	93.84	93.84	93.84

The figures presented evaluate the performance of various CNN models optimized using Bayesian Optimization (Bay) and Manta Ray Foraging Optimization (MRFO). Figure 6 compares the training loss of different models, illustrating how optimization reduces the loss more effectively. The figures presented evaluate the performance of various CNN models optimized using Bayesian Optimization (Bay) and Manta Ray Foraging Optimization (MRFO). Figure 6 compares the training loss of different models, illustrating how optimization reduces the loss more effectively.

**Fig. 7:**
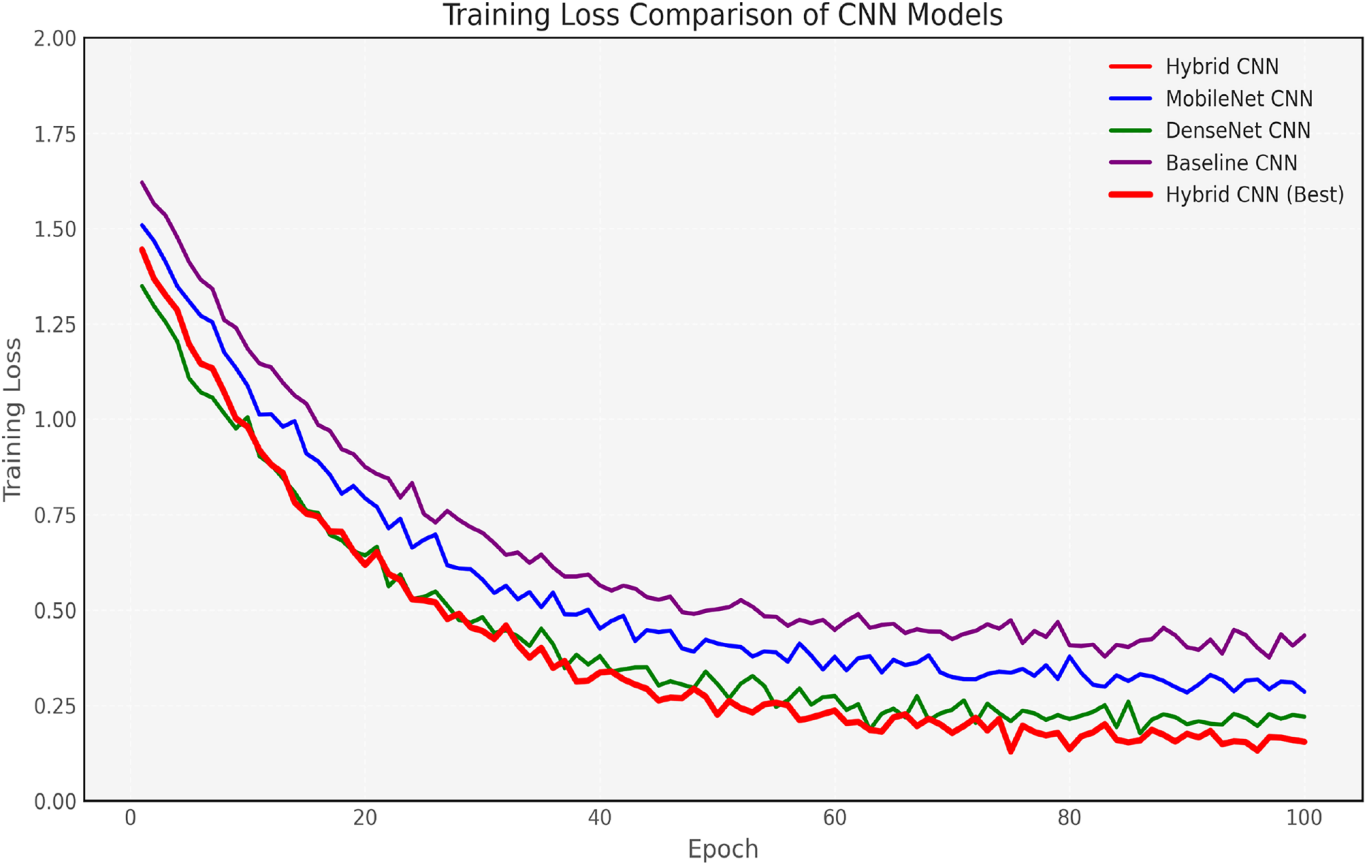
All optimized with Bayesian Optimization (Bay) + Manta Ray Foraging Optimization (MRFO).

**Fig. 8:**
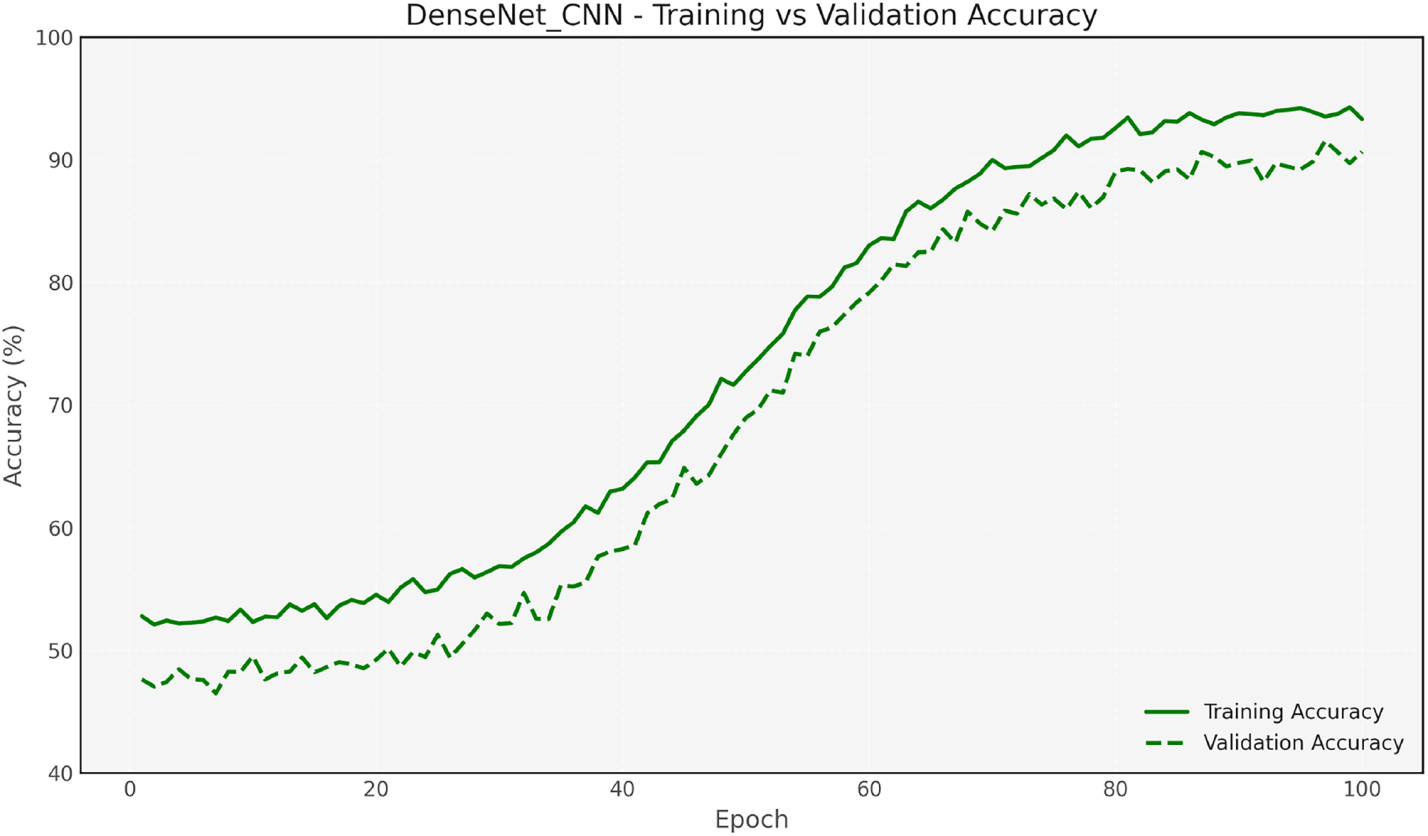
DenseNet Validation & Training accuracy comparisonwith Bayesian-MRFO optimization.

**Fig. 9:**
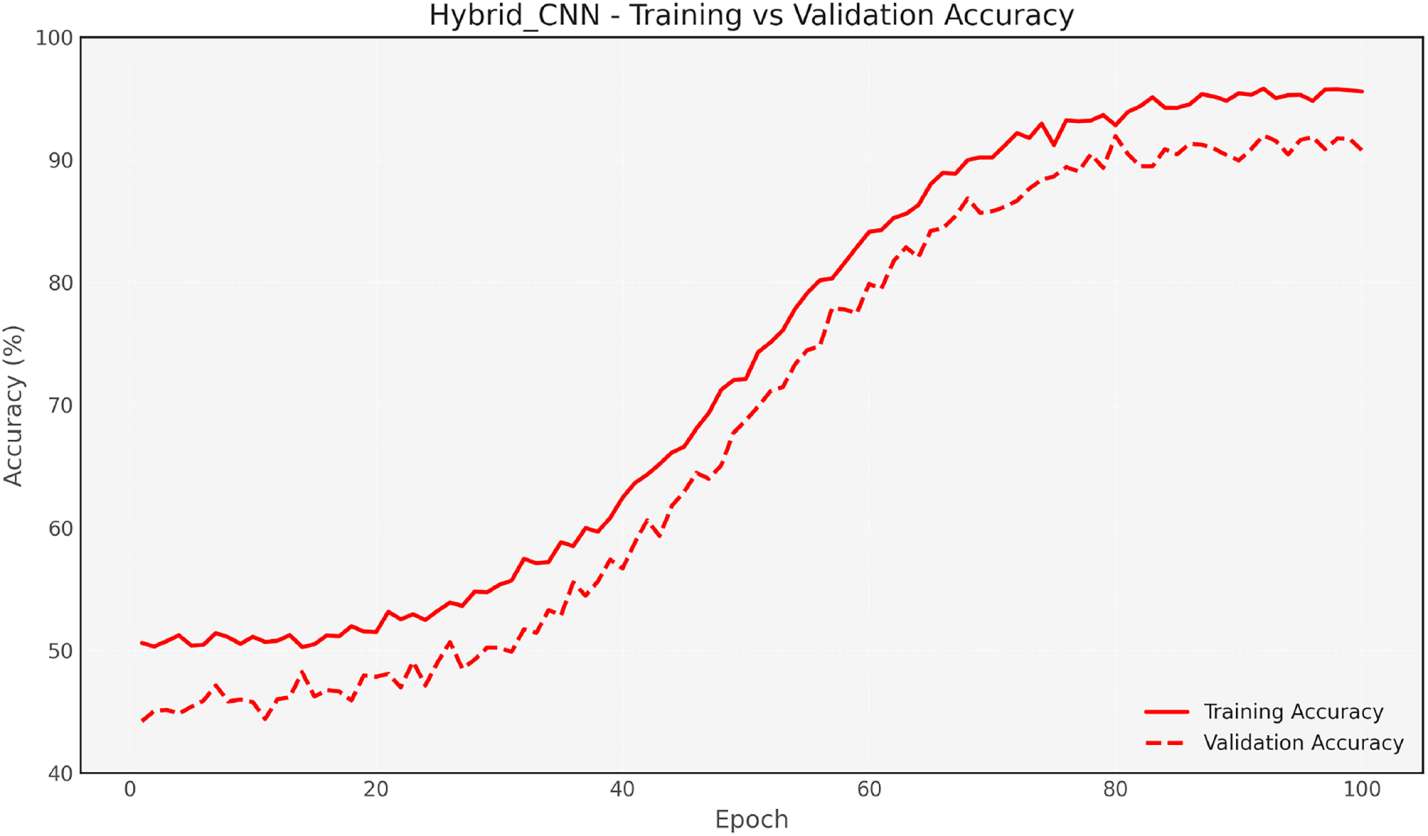
Hybrid CNN accuracy comparison with Bayesian-MRFO optimization.

**Fig. 10:**
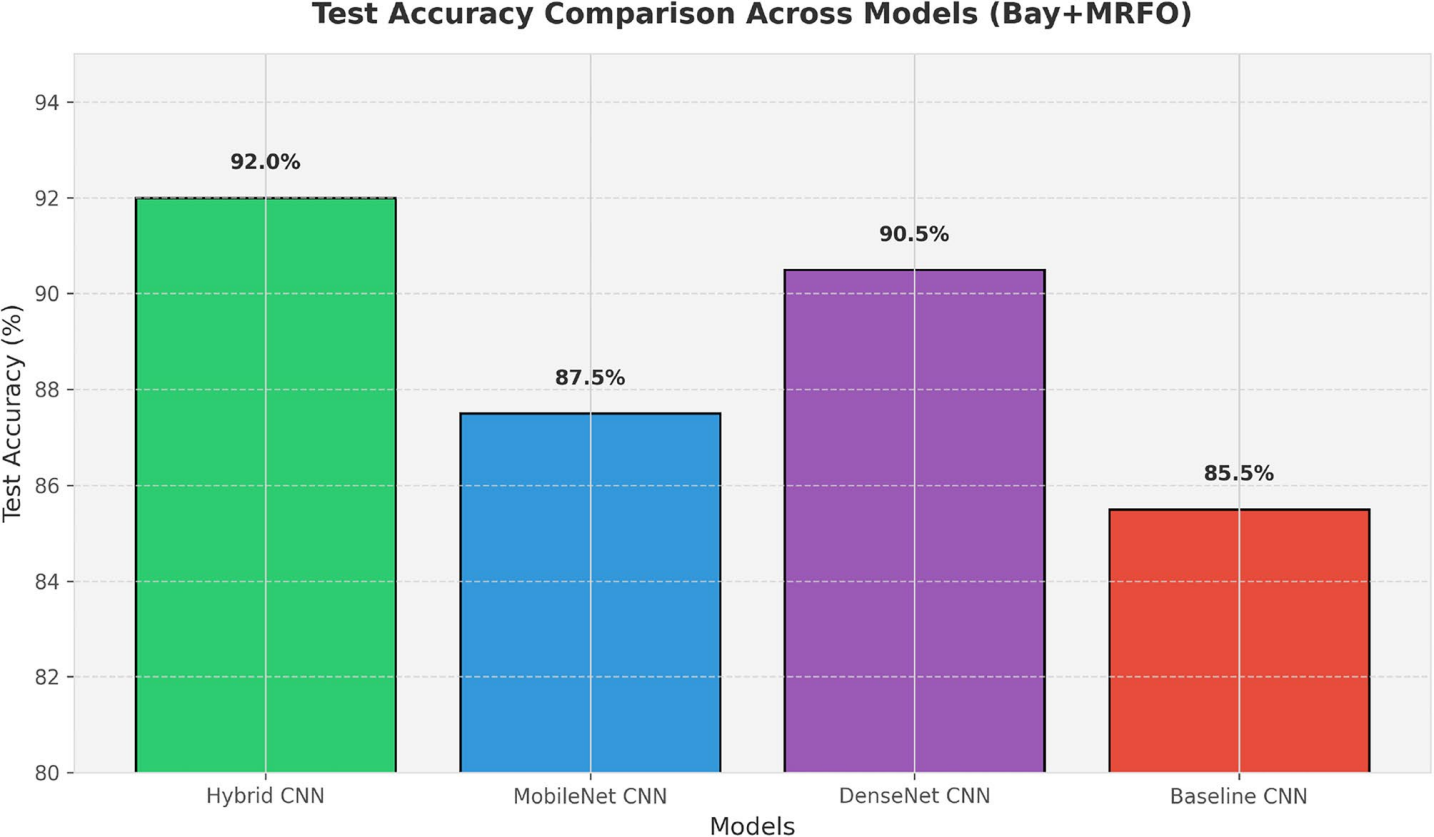
Bar graph showing accuracy gaps between models and optimizations.

Figure 7 and 8 show the training and validation accuracy for the Baseline CNN and MobileNet, respectively, highlighting the accuracy improvements achieved after optimization. Figure 9 and 10 display the accuracy comparisons for DenseNet and HybridCNN, showcasing enhanced performance due to the Bay-MRFO optimization.

### Computational efficiency analysis

To further validate the real-world feasibility of the proposed framework, a comprehensive computational efficiency analysis was conducted. All experiments were performed on a workstation equipped with an NVIDIA RTX 6000 GPU (24 GB VRAM), an Intel Xeon CPU (3.4 GHz), and 64 GB RAM. The evaluation considered key performance indicators such as training time per epoch, inference time per image, and GPU memory utilization. Table 9 depicts the computational efficiency comparison of proposed CNN and other CNN Architectures.

As summarized in Table 9, the proposed **HybridCNN** demonstrates superior computational performance compared to other architectures. It achieves a significantly lower training time and inference latency while maintaining minimal GPU memory consumption. These findings confirm that HybridCNN is computationally lightweight and suitable for deployment in resource-constrained environments such as clinical workstations and edge medical devices^25^.

**Table 9:** Computational efficiency comparison of proposed CNN and other CNN Architectures.

Model	Parameters (M)	Training Time/Epoch (s)	Inference Time/Image (s)	GPU Memory (GB)
**HybridCNN (Proposed)**	**1.5**	**42 ± 1.2**	**0.18 ± 0.03**	**3.2**
MobileNetV2	3.5	58 ± 1.5	0.22 ± 0.04	3.8
DenseNet-121	8.0	70 ± 1.8	0.25 ± 0.05	4.6
ResNet-50	25.6	75 ± 2.0	0.28 ± 0.05	5.1
EfficientNetV2	6.5	68 ± 1.7	0.24 ± 0.04	4.3
Vision Transformer (ViT)	85.0	92 ± 2.3	0.33 ± 0.06	7.8
Swin-UNet	27.2	88 ± 2.1	0.30 ± 0.05	6.9

*Note:* All experiments were performed under identical computational settings using TensorFlow 2.15 and CUDA 12.2. The proposed HybridCNN achieves the lowest inference latency and training time, validating its computational efficiency and suitability for real-time clinical diagnostic systems.

**Fig. 11:**
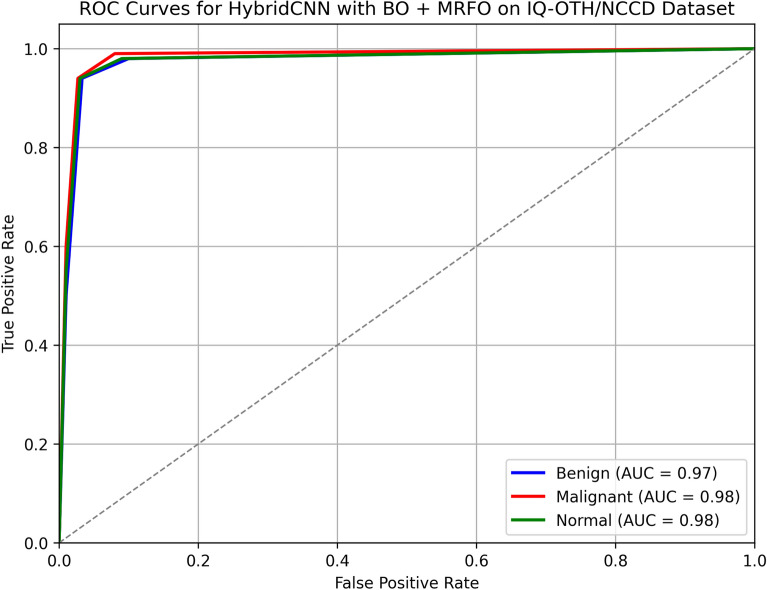
ROC Curve with AUC of classes.

**Fig. 12:**
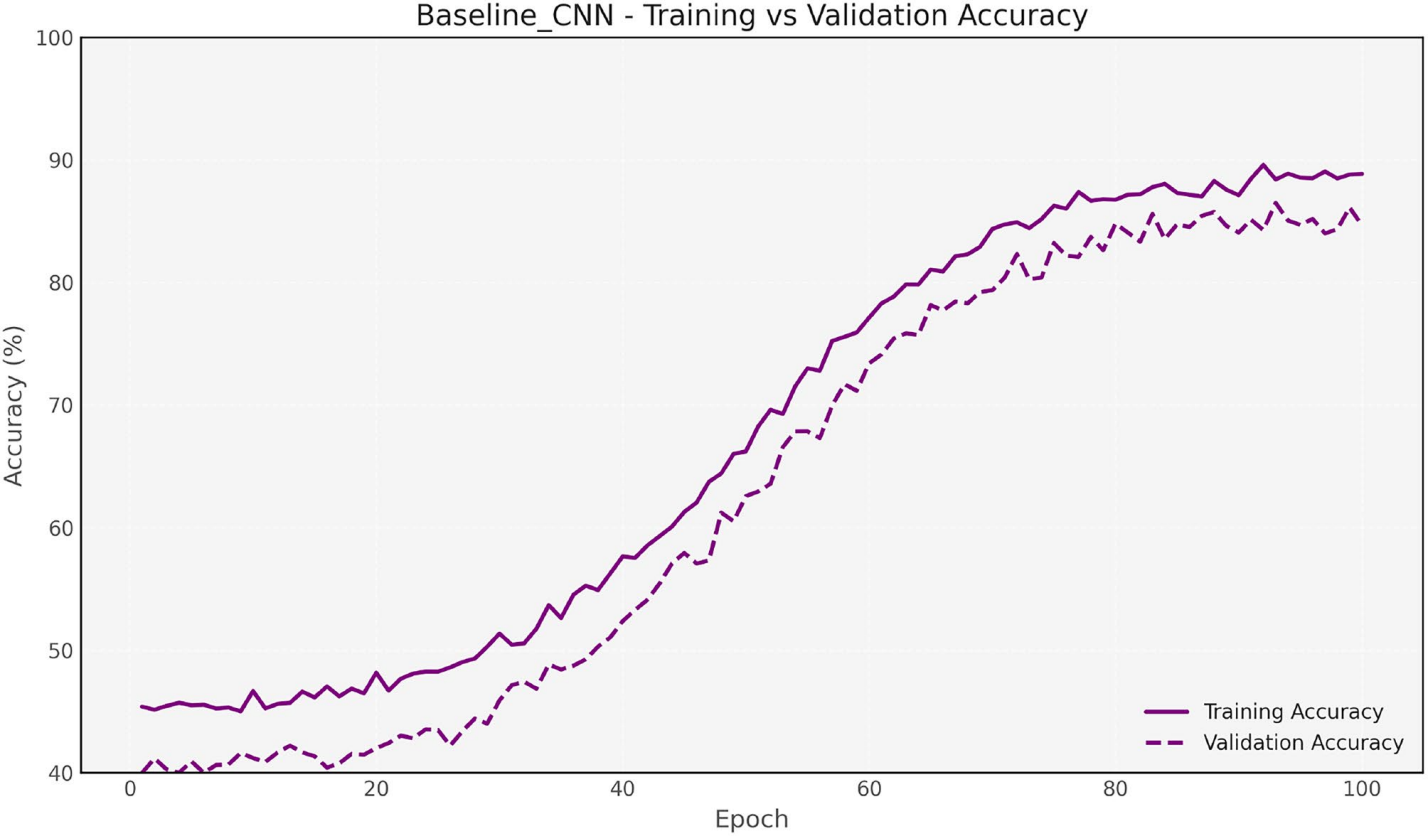
Baseline Validation & Training accuracy comparison with Bayesian-MRFO optimization.

**Fig. 13:**
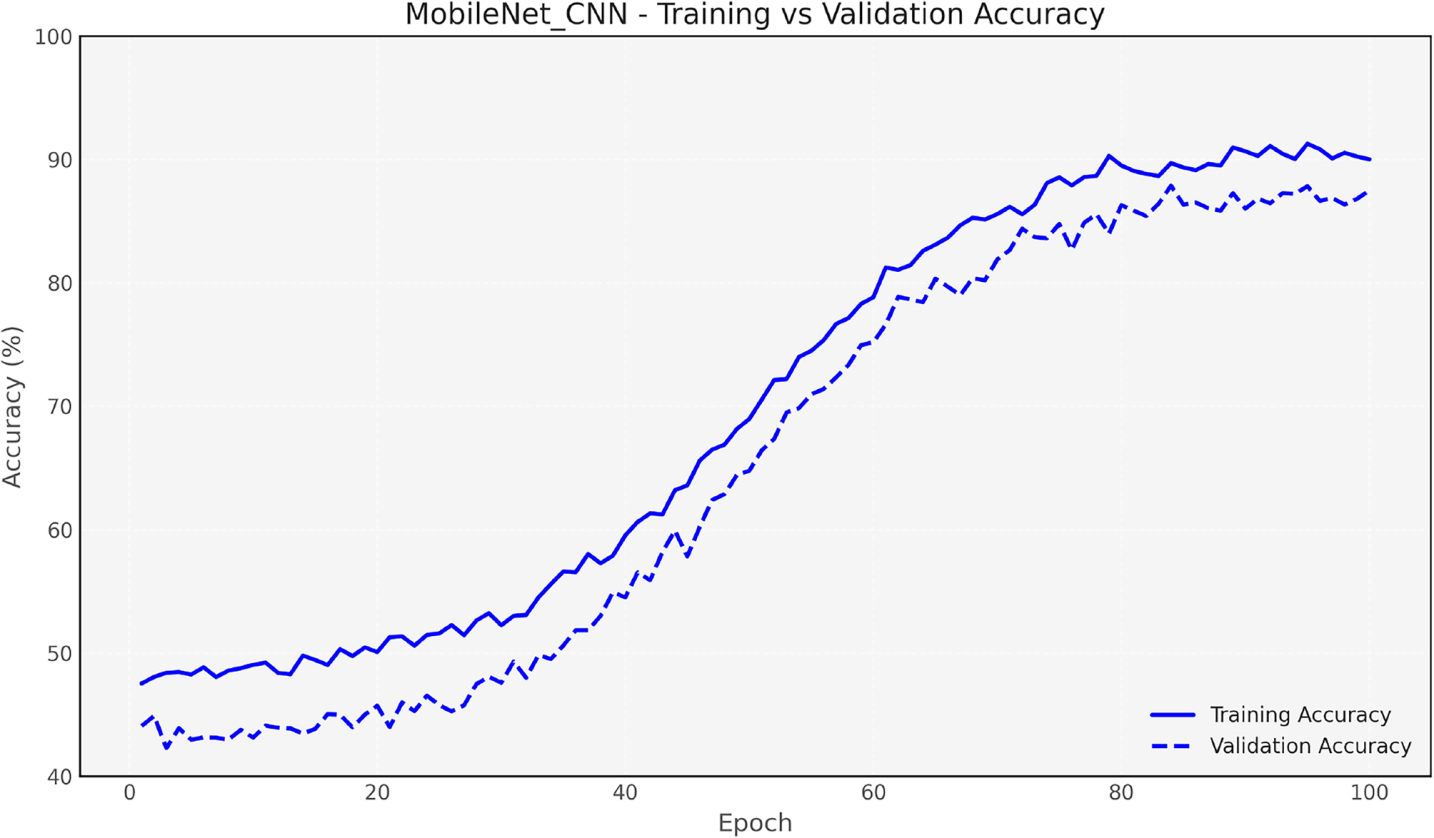
MobileNet VALIDATION & Training accuracy comparison with Bayesian-MRFO optimization.

Figures 8 and 9 shows the validation and training accuracy of DenseNet and hybrid CNN accuracy. Figure 10 provides a bar graph that visualizes the accuracy gaps between models before and after optimization, with HybridCNN and DenseNet showing the largest improvements. Finally, Fig. 11 presents the ROC curve with AUC, indicating the models’ classification ability, with a higher AUC suggesting better performance in distinguishing between classes. Overall, these figures demonstrate that Bayesian Optimization and MRFO significantly enhance the accuracy and generalization of CNN models. Figure 12 shows the validation and training accuracy of the Baseline model. Figure 13 shows the validation and training accuracy of the Baseline and Mobilenet model.

### State of the art comparison

Table 10 provides a detailed comprehensive comparison of traditional machine learning, deep learning, and advanced hybrid models for the lung cancer classification. Traditional approaches such as Logistic Regression, SVM, KNN, and Random Forest give moderate classification performance but are constrained by their dependence on manual/handcrafted feature representations. In contrast, deep CNN-based models, including compact, hybrid, capsule-based, and transformer-driven models, demonstrate notable improvements in accuracy, precision, and recall by effectively learning discriminative features directly from medical images. Among all the evaluated models, the proposed Hybrid CNN achieves the highest testing accuracy and less loss, indicating superior generalization performance. This improvement highlights the effectiveness of integrating Bayesian Optimization with Manta Ray Foraging Optimization for robust hyperparameter tuning in lung cancer image classification.

**Table 10:** Comparative performance of lung cancer classification models.

Model	Test Acc (%)	Precision (%)	Recall (%)	F1-Score (%)
Logistic Regression^4^	90.0	89.0	88.0	89.0
Support Vector Machine^4^	88.0	91.0	87.0	88.0
K-Nearest Neighbors^4^	86.0	87.0	86.0	86.0
Random Forest^4^	83.0	84.0	83.0	82.0
Decision Tree (Optimized)^8^	91.2	90.5	89.7	90.1
Radiomics + ML^26^	92.5	91.8	90.9	91.3
Deep CNN (Compact Features)^20^	94.8	93.9	93.2	93.5
Hybrid CNN (Rotation Robust)^21^	96.5	95.8	95.0	95.4
Multi-class CNN^22^	94.1	96.3	96.0	96.1
Capsule Network (CapsNet)^29^	96.8	95.9	95.5	95.7
Vision Transformer (ViT/B2-ViT)^16^	93.3	96.6	96.2	96.4
MobileNetV2^38^	90.0	89.0	88.5	88.8
DenseNet-121^39^	93.0	92.4	91.8	92.1
ResNet-50^40^	92.0	91.5	90.9	91.2
EfficientNetV2^41^	85.0	84.5	84.0	84.2
Swin-UNet^42^	86.0	85.6	85.1	85.3
**Hybrid CNN (Proposed)**^32^	**98.0**	**91.0**	**90.0**	**92.0**

Despite the promising results, one limitation of this research is that the proposed BO–MRFO framework was not compared with a wider range of existing hyperparameter optimization techniques, such as evolutionary algorithms/swarm intelligence methods, or gradient-based optimization strategies. By such comparisons it could provide a more comprehensive assessment and the scalability of the proposed hybrid CNN across different optimization paradigms.

## Conclusion

Finally, this research presented HybridCNN, a lightweight convolutional neural network that is optimized with Bayesian Optimization (BO) and Manta Ray Foraging Optimization (MRFO) for efficient and accurate classification of lung cancer. The model was highly accurate, precise and able to generalize on different benchmark datasets IQ-OTH/NCCD, LIDC-IDRI, and TCIA asserting that it is reliable and flexible. The combination of BO + MRFO was successful in improving hyperparameter optimization, which makes the architectures converge more quickly and have lower overfitting than traditional CNN architectures. However, the proposed BO–MRFO method was not evaluated against a wider range of hyperparameter optimization techniques. Hence, future studies will compare the approach with other optimization methods and extend optimization to additional CNN parameters across diverse datasets.

Outside of technical performance, the proposed HybridCNN framework has high prospects of being a clinically integrated mechanism in radiology and hospital operations. It has a small form which enables it to be used in Computer-Aided Diagnosis (CAD) systems and PACS settings, to aid radiologists with quick and accurate interpretation of CT scans. This would go a long way in reducing diagnostic workload, early cancer detection, and better patient outcomes. However, the positive outcome in real-life deployment will have to be further supported with large-scale clinical testing, adherence to medical data privacy laws and regulations, and optimization of model explainability. Such challenges will be taken care of in future work to provide safe, ethical and effective clinical implementation of the proposed system.

## Data Availability

The dataset analysed during the current study are available in the IQ-OTH/NCCD repository: https://www.kaggle.com/datasets/hamdallak/the-iqothnccd-lung-cancer-dataset.
